# Two members of the DUF579 family are responsible for arabinogalactan methylation in Arabidopsis

**DOI:** 10.1002/pld3.117

**Published:** 2019-02-12

**Authors:** Henry Temple, Jenny C. Mortimer, Theodora Tryfona, Xiaolan Yu, Federico Lopez‐Hernandez, Mathias Sorieul, Nadine Anders, Paul Dupree

**Affiliations:** ^1^ Department of Biochemistry University of Cambridge Cambridge UK

**Keywords:** arabinogalactan proteins, Golgi apparatus, methyltransferases, polysaccharide synthesis

## Abstract

All members of the DUF579 family characterized so far have been described to affect the integrity of the hemicellulosic cell wall component xylan: GXMs are glucuronoxylan methyltransferases catalyzing 4‐O–methylation of glucuronic acid on xylan; IRX15 and IRX15L, although their enzymatic activity is unknown, are required for xylan biosynthesis and/or xylan deposition. Here we show that the DUF579 family members, AGM1 and AGM2, are required for 4‐O–methylation of glucuronic acid of a different plant cell wall component, the highly glycosylated arabinogalactan proteins (AGPs).

Plant cells are surrounded by a complex extracellular matrix composed primarily of polysaccharides, glycoproteins and in certain tissues lignin. Type II arabinogalactans (AGs) are large complex branched polysaccharide structures, attached to the hydroxyproline residues of many plant cell wall polypeptides. Proteins attached to AGs are defined as arabinogalactan proteins, and these proteins form a diverse class of cell surface proteoglycans found in most plant species throughout the plant kingdom (Showalter, 2001). AGPs play a role in several biological processes, such as cell adhesion, development, wound healing and salt or drought resistance (Seifert & Roberts, 2007). The type II AG polysaccharide structure is very heterogeneous, but commonly consists of a β‐1,3‐galactan backbone with substitution at the O6 position with β‐1,6‐galactan side chains (Tan et al., 2012). β‐1,6‐galactan side chains, which may have β‐1,3‐kinks, are frequently substituted by arabinose (Ara) and occasionally substituted with other sugars such as rhamnose or fucose. The β‐1,6‐galactan side chains often terminate with β‐1,6‐glucuronic acid (GlcA) or 4‐*O*‐methyl glucuronic acid (MeGlcA). In addition, glucuronylation can also take place at the O6 of the β‐1,3‐galactan backbone (Tryfona et al., 2012). The AGP glycosylation occurs in the Golgi apparatus by the concerted action of different glycosyltransferases (GTs), and it has been proposed that at least ten GT activities are required for type II AG biosynthesis (Knoch, Dilokpimol, & Geshi, 2014). To date several glycosyltransferases have been described to function in AG synthesis. Among these activities, three members of the GT14 family have been reported to have β‐1,6‐glucuronyltransferase activity (GlcAT14A, GlcAT14B, GlcAT14C). The biological role of GlcA and MeGlcA modifications on AGPs is unclear. However, a mutant in GlcAT14A was reported to have a seedling growth phenotype (Knoch et al., 2014). While GlcA on AGs is frequently 4‐*O*‐methylated (Tryfona et al., 2012), to date an AGP *O*‐methyltransferase has not been identified.

DUF579 family members of higher plants are clustered in four clades (Figure 1a). The three members of the DUF579 family Clade I, GXM1‐3, are 4‐*O*‐methyltransferases using S‐adenosylmethinonine as donor to catalyze the transfer of 4‐*O*‐methyl to glucuronic acid on xylan (Lee et al., 2012; Li et al., 2013; Urbanowicz et al., 2012). In addition, two DUF579 members of Clade II, IRX15 and IRX15L, are implicated in xylan biosynthesis and/or deposition of xylan in secondary cell walls (Brown et al., 2011; Jensen et al., 2011). *irx15 irx15l* double mutants show disorganized secondary cell walls and an increase in the amount of sugar released in saccharification assays. The function of Clade III and Clade IV family members is unknown. Interestingly, all four clades seem not to comprise representatives of lower plants, as Physcomitrella and Selaginella genes group distinctly, suggesting specialization of DUF579 proteins through the evolution of land plants into different clades.

**Figure 1:**
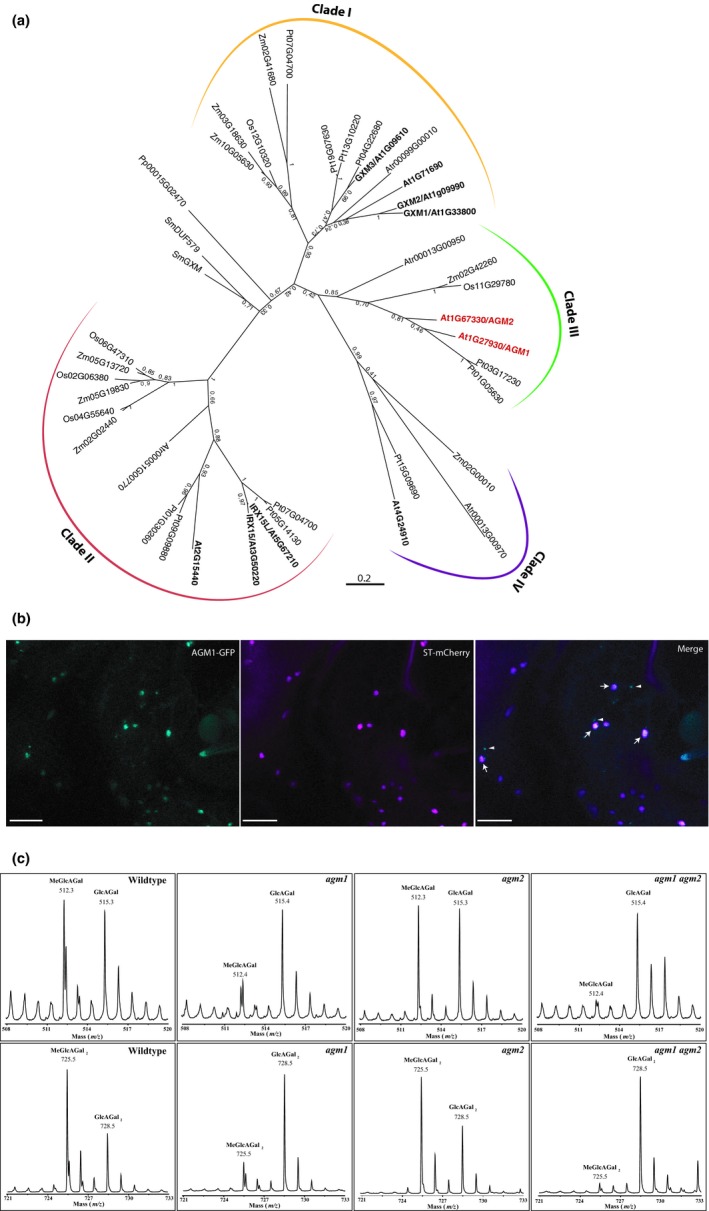
Members of DUF579 are required for arabinogalactan glucuronic acid methylation. (a) Phylogenetic tree of DUF579 proteins of *Arabidopsis thaliana* (At), *Amborella trichopoda* (Atr), *Oryza sativa* (Os), *Physcomitrella patens* (Pp), *Populus trichocarpa* (Pt), *Selaginella moellendorffii* (Sm) and *Zea mays* (Zm). Arabidopsis proteins in bold, AG glucuronic acid methyltransferases (AGM1 and AGM2) are highlighted in red. (b) Sub‐cellular localization of AGM1. Tobacco epidermal cells were co‐transformed with *Agrobacteria* carrying vectors containing *AGM1‐GFP* and the Golgi marker *ST‐mCherry*. Note: AGM1 colocalizes with ST in Golgi stacks (arrows), no colocalization in small punctate structures (arrow heads). (c) MALDI‐ToF‐MS analysis of oligosaccharides released after root AG digestion using AG specific enzymes, α‐l‐arabinofuranosidase and exo‐β(1→3)‐galactanase of Wildtype, *agm1*,* agm2* and *agm1 agm2* double mutant plants. Oligosaccharides were deuteropermethylated, giving a mass difference of 3 Da between the oligosaccharides with MeGlcA versus GlcA (Ion mass shown is [M+Na]^+^). Upper panel: [Me]GlcAGal, lower panel [Me]GlcAGal_2_

GXM and IRX15 proteins localize to the Golgi apparatus. Localization of Organelle Proteins by Isotope Tagging (LOPIT) proteomics data suggested that At1g27930 (termed AGM1 hereafter), an Arabidopsis Clade III DUF579 family member, is also a Golgi localized protein (Nikolovski et al., 2012). To confirm the proteomics data, and investigate its sub‐cellular localization more closely, we transiently expressed AGM1 fused to GFP in tobacco leaves. AGM1‐GFP co‐localized with the Golgi marker ST‐RFP, confirming the localization in the Golgi apparatus (Figure 1b). Additional small punctate signals were also identified, as previously noted for IRX15 proteins (Brown et al., 2011).

AGM1 and AGM2 (At1g67330) share 38–42% identity with the glucuronoxylan 4‐O‐methyltransferases (GXMs) which are their closest DUF579 paralogs in Arabidopsis (Supporting information Table S1). To assess whether Clade III DUF579 proteins are involved in xylan biosynthesis, as observed for Clade I and II members, we identified knock‐out T‐DNA insertion lines of both Arabidopsis Clade III genes, *At1g27930* (*AGM1*) and *At1g67330 (AGM2)* (Supporting information Figure S1) and analyzed the xylan structure of *agm1*,* agm2* and *agm1 agm2* double mutants, using Xyn11A xylanase fingerprinting by DNA‐sequencer Assisted Saccharide analysis in High‐throughput (DASH). Although both genes are expressed in roots according to the AtGeneExpress data (Supporting information Figure S2), we did not detect obvious changes in root xylan GlcA methylation in the single and double mutants (Supporting information Figure S3).

We next investigated whether *agm1*,* agm2* and *agm1 agm2* double mutants show reduced methylation of the GlcA in another cell wall component, namely the AGPs. Extracted AGPs from roots were treated with the AG specific enzymes α‐L‐arabinofuranosidase and exo‐β(1→3)‐galactanase to cleave the backbone, and the perdeuteromethylated oligosaccharides were analyzed using MALDI‐ToF‐MS. In Wild‐type AG the released oligosaccharides, [Me]GlcA‐β–1,6‐Gal and [Me]GlcA‐β‐1,6‐Gal‐β‐1,6‐Gal (Hereafter simplified to [Me]GlcAGal and [Me]GlcAGal2), were highly methylated (Figure 1c, [Me] denotes methylated and unmethylated oligosaccharides). On the contrary, in the *agm1* mutant, methylation of GlcA in both oligosaccharides was greatly reduced. This decrease in AGP GlcA methylation could be restored by expressing a FLAG‐tagged version of AGM1 under the native promoter in the *agm1* mutant background (Supporting information Figure S4). Although *AGM2* mutation alone did not cause a reduction of AGP MeGlcA, the absence of expression of both *agm1* and *agm2* resulted in the absence of AG GlcA methylation in the *agm1 agm2* double mutant (Figure 1c). This shows that both AGM proteins are required for methylation of root AGPs. Given these results and considering the homology to GXMs, our data strongly suggest that Clade III genes, *agm1* and *agm2*, encode the GlcA *O*‐methyltransferases for AGPs, hence we named the proteins ArabinoGalactan Methyltransferases (AGMs). The oligosaccharides GlcA‐Gal and GlcA‐Gal_2_ derive from the backbone and side chains, respectively (Tryfona et al., 2012). Since methylation of both GlcA‐Gal and GlcA‐Gal_2_ was reduced in the *agm1* single mutant and double mutants, AGM1 and AGM2 are both involved in methylation of GlcA on both AG structures in roots. Given the widespread expression of AGM1 (Supporting information Figure S2), it is likely that it is responsible for methylation of AG in other tissues.

Our results show that the 4‐*O*‐methyl transfer activity onto GlcA is likely conserved between Clade I and III. Interestingly, *Selaginella moellendorffii* xylan GlcA is almost completely methylated and one of the two DUF579 members in *Selaginella* has been reported to have GXM activity (Haghighat, Teng, Zhong, & Ye, 2016). Taken together, these data suggest that the methyltransferase activity might be conserved in the other Clades, Clade II (IRX15) and Clade IV of the DUF579 family. However, they may methylate different polysaccharides or may methylate GlcA in xylan or AG in different tissues or in specific polysaccharide contexts, such as in APAP1 (arabinoxylan pectin arabinogalactan protein1, Tan et al., 2013). Methylated GlcA has not been found on *Physcomitrella patens* xylan (Kulkarni et al., 2012) suggesting the single *P. patens* DUF579 may methylate GlcA of a different polysaccharide. It would be interesting to test whether this activity is similar to the AGMs activities described in this work. Alternatively, uncharacterized DUF579 members could be involved in methylation of other sugars, for example 3‐O‐methylation of rhamnose in AG or the 2‐O‐methylation of xylose or fucose present in rhamnogalacturonan II.

In our growth conditions *agm1 agm2* did not display any obvious growth phenotypes, showing that AG methylation is not essential for viability. It has been also reported that some AGP mutants show a hypocotyl length phenotype, but we did not detect differences in hypocotyl length in etiolated plants (Supporting information Figure S5). While we do not observe obvious fertility differences in these mutants, it has recently been shown that AG provides a signaling molecule in pollen tube guidance and the methyl group of GlcA in the AG is critical for the effectiveness of the signal (Mizukami et al., 2016). There are other potential roles for the methyl group on GlcA. For example, GlcA on AGs can chelate calcium ions (Lamport & Varnai, 2013). 4‐*O*‐methylation of GlcA would change the calcium binding affinity, thus modulating the calcium release response to pH. Moreover, the addition of the methyl group to GlcA prevents the addition of 4‐linked sugars, such as rhamnose, and will also prevent extension of 4‐linked side chains to the GlcA of AG as seen in APAP1 (Tan et al., 2013). Therefore, modulating the AG structure through the activity of AGMs may provide a means of adapting the AG structure to diverse physiological processes.

## CONFLICT OF INTEREST

The authors declare no conflict of interest associated with the work described in this manuscript.
