# Supplementary material for: Two members of the DUF579 family are responsible for arabinogalactan methylation in Arabidopsis
Source: Plant Direct. 2019 Feb 12;3(2):e00117. doi: 10.1002/pld3.117 (PMC6508755; doi:10.1002/pld3.117)
Supplement: Supplementary file 1 [file PLD3-3-e00117-s001.docx]

**Supplemental Materials**

**Material and Methods**

**Plant material and growth conditions**

All Arabidopsis T-DNA insertion mutant lines were in Col-0 ecotype. SALK_000253 (*agm1-1*), GABI_054A04 (*agm2-1)* and SALK_057182 (*agm2-2*) seeds were obtained from the European Arabidopsis Stock Centre (NASC, Nottingham, UK). Seeds were surface sterilized and sown on 0.8% (w/v) agar, 0.5x Murashige and Skoog salts including vitamins (Sigma) and sucrose (1% w/v). Following stratification for 48h at 4ºC in the dark, plates were transferred to a growth room (20ºC, 100 μmol m^-2^ sec^-1^, 24h light, 60% humidity), plates were placed vertically for 12 days and then plants were transferred to hydroponic media ([Gibeaut et al., 1997](#_ENREF_2)). Tissue used to isolate AGPs from roots was collected from six week old plants grown hydroponically frozen in liquid nitrogen and stored in -80°C. Arabidopsis leaf and root AGPs were prepared using a previously established protocol ([Tryfona et al., 2012](#_ENREF_10)).

**Sequence analysis and phylogeny**

Amino acid sequences of the species *Amborella trichopoda*, *Arabidopsis thaliana*, *Oryza sativa*, *Physcomitrella patens*, *Populus trichocarpa*, *Zea mays*, were retrieved from PLAZA 3.0 dicots database (http://bioinformatics.psb.ugent.be/plaza/versions/plaza_v3_dicots/) ([Proost et al., 2015](#_ENREF_5)). Due incomplete amino acid sequences and truncated DUF579 domain the following sequences were not included in the analysis: Atr_00050G00490, Atr_00122G00500, Os02G27140, Os05G37530, Pt12G09810, Zm01G12200, Zm02G02460, Zm02G11810. Sequences included of *Selaginella moellendorffii* were named as described ([Haghighat et al., 2016](#_ENREF_3)), taken from GenBank (SmGXM GenBank: KX372532) and GenPept (SmDUF579 XP_002983636) (https://www.ncbi.nlm.nih.gov/). Sequences were aligned using MUSCLE (**MU**ltiple **S**equence **C**omparison by **L**og-**E**xpectation), and the phylogenetic analysis was obtained with the neighbour-joining method with bootstrap values generated from 500 bootstrap samples was generated using Molecular Evolutionary Genetics Analysis (MEGA) 7 and visualized by FigTree 1.4.2.

**Identification of homozygous knock-out T-DNA insertion mutants**

For the identification of *agm1* knock-out plants, RNA from 4 weeks old rosette leaves of Col-0, *agm1.1* and *agm1.2* plants were prepared using Qiagen RNeasy plant mini kit according to instructions of manufacturer. cDNA was synthesised using Superscript II^TM^ Reverse Transcriptase from Thermo Fisher using Oligo dT according to instructions of manufacturer. For identification of *agm2* knock-out plants, RNA was prepared from roots of 2 weeks old Col-0 and *agm2* plants grown in 0.5x Murashige and Skoog plates using Qiagen RNeasy plant and cDNA synthesis was performed as described above. *agm1.1* and *agm1.2*. RT-PCR experiments were performed with the cycles and primers described in Table S2. GADPH was used as housekeeping. Only *agm1.2* is a knock-out line, all further experiments were done only using *agm1.2* disregarding *agm1.1*. For simplicity *agm1.2* is named *agm1* in the main text. Primers used for RT-PCR experiments are the following: *AGM1* was amplified using the ptimers 5’ AACGCTCTTCCTCTGTTCCA 3’ and CACGAGATGCAGATCCAAAA 3’, *AGM2* was amplified using the primers 5’ CACCTTGTCACTCTGCTCCA 3’ and 5’ TGATGGCCGAGGAAAATATC 3’, *GAPDH* was amplified using the primers 5’ TGCAATCCCAGCCTTGGCATG 3’ and 5’ TAACTGCCTTGCTCCTCTTGCCCAAG 3’.

**Cloning procedures**

For *AGM1* sub-cellular localisation *AGM1* CDS sequenced flanked by the sites XbaI and SalI was inserted into pVKH18en6 vector ([Sparkes et al., 2006](#_ENREF_8)). For *AGM1-FLAG* genetic complementation, intergenic region was used as promoter and coding sequences of *AGM1* was fused C-terminally with the FLAG tag using a modified version of pGreenII vector.

**Subcellular localisation**

Six-week-old tobacco leaves were infiltrated with Agrobacterium strain GV3101:pMP90 as described ([Sparkes et al., 2006](#_ENREF_8)). Two independent transformations were used for analysing AGM1 subcellular localisation. Golgi co-localisation was confirmed by using the Golgi Marker Sialyl Transferase (ST) ([Wee et al., 1998](#_ENREF_12)) fused to mCherry. Fluorescent signals were analysed 60 h after infiltration by confocal laser scanning microscopy using a Leica TCS SP8 confocal microscope.

**AGP enzymatic hydrolysis**

Arabinogalactan preparations (1 mg) were digested with the AG-specific enzymes as described ([Tryfona et al., 2010](#_ENREF_9)). Briefly, AGP extracts were digested with recombinant α-L-arabinofuranosidase in 50 mM ammonium acetate buffer (pH 5.0, 100 μl) at 37 ˚C for 24 h. The enzyme was inactivated for 5 min at 100 ˚C and the sample was dried in a rotary evaporator. An exo-β-(1→3)-galactanase in 20 mM ammonium acetate (pH 4.6, 100 μl) followed at 37 ˚C for 24 h.

**AGP oligosaccharide sample desalting and clean-up**

Following the enzyme digestions and prior to *per*-methylation, released peptides and enzymes were removed using reverse-phase Sep-Pak C_18_ cartridges (Waters) as described previously ([Tryfona et al., 2010](#_ENREF_9)). Briefly, the AG oligosaccharides were eluted with 3 ml of 5% acetic acid and were lyophilised. Dry samples were dissolved in 0.5 ml of 5% acetic acid and were desalted using 2 ml Dowex beads (50 x 8, H^+^ form, 50-100 mesh). Purified samples were lyophilised.

**Xylan fingerprinting using DNA sequencer-Assisted Saccharide analysis in High throughput (DASH)**

DASH experiments were performed as described ([Li et al., 2013](#_ENREF_4)). Briefly, roots cell wall material was digested using *Neocallimastix patriciarum* Xyn11A xylanase at 21°C. Released oligosaccharides were derivatised with APTS, diluted and analysed using an ABI 3730xl DNA sequencer.

**Perdeuteromethylation of arabinogalactan polysaccharides**

Perdeuteromethylation of glycans was performed using the NaOH slurry method described by Ciucanu and Kerek ([Ciucanu and Kerek, 1984](#_ENREF_1)) using 1 ml of deuteromethyl iodide (Fluka). Dry samples were resuspended in 100 μl MeOH and were kept at room temperature for MALDI-ToF/ToF-MS/MS analysis.

**MALDI-ToF-MS**

Perdeuteromethylated samples were analysed by MALDI-ToF-MS (4700 Proteomics Analyser, Applied Biosystems, Foster City, CA, USA) and a Bruker Ultraflextreme, as previously described ([Tryfona and Stephens, 2010](#_ENREF_11)), using 2,5-dihydroxybenzoic acid (2,5-DHB) matrix (20 mg ml^-1^ dissolved in 50% MeOH). Data were acquired using a 200 Hz frequency triple Nd-YAG laser operating at 355 nm wavelength (4700 tandem mass spectrometer) or 2-kHz smart beam II laser (UltrafleXtreme) in reflector mode (mass range 300-3000 Da).

**Etiolated hypocotyl growth**

Sterilised seeds of Arabidopsis Col-0, *agm1*, *agm2* and *agm1 agm2* were sown on 1x Murashige and Skoog plates as described above, stratified for 72h at 4ºC in the absence of light, after stratification plates were exposed to light for 4h at 21ºC and then covered with aluminium foil and placed vertically. After 9 days of etiolated growth, plates were scanned and hypocotyl growth was measured using ImageJ software ([Schneider et al., 2012](#_ENREF_7)).

**Supplemental Figures**

**Supplemental Figure 1. Identification of *agm1* and *agm2* T-DNA insertion knock out mutants. (A)** Gene model representing *AGM1* and *AGM2*. Red triangles represent the position of T-DNA insertion in mutant lines. Blue arrows represent the primer alignment position used for RT-PCR experiments. **(B)** Analysis of *AGM1* and *AGM2* expression in Wildtype (Wt) and T-DNA insertion lines. RT-PCR experiments to detect presence of *AGM1* and *AGM2* transcripts in wildtype, *agm1* and *agm2*. *GAPDH* transcript amplification was used as a control. Note: *agm2.1* is not a knock-out and was not used in further analysis.

**Supplemental Figure 2. Expression pattern of *AGM1* and *AGM2* in *Arabidopsis thaliana* development.** Absolute signal values of specific expression points obtained from Affimetrix ATH1 arrays, displayed in AtGenExpress ([http://jsp.weigelworld.org/AtGenExpress/)](http://jsp.weigelworld.org/AtGenExpress/resources/)) ([Schmid et al., 2005](#_ENREF_6)) were chosen to analyse *AGM1* and *AGM2* expression in different tissues and developmental stages.

**Supplemental Figure 3. Analysis of xylan 4-*O* methyl glucuronic acid in *agm* mutants.** Xylan was digested with xylanase NpXyn11A, and analysed by DASH capillary electrophoresis. Note, the frequency of xylan GlcA substitution and the extent of methylation of GlcA were not substantially altered in roots of the *agm* single and double mutants compared to Wildtype.

**Supplemental Figure 4.** ***agm1* rescued lines restore the levels of MeGlcA in AGPs.** MALDI-ToF-MS analysis of oligosaccharides released after AG digestion using AG specific enzymes, α-L-arabinofuranosidase and exo-β-(1→3)-galactanase of AG from Wildtype, *agm1* and AGM1-FLAG in *agm1* plants. Oligosaccharides were perdeuteromethylated, giving a mass difference of 3 Da between the oligosaccharides with MeGlcA vs GlcA (ion mass shown is [M+Na]^+^). Upper panel: [Me]GlcAGal, lower panels [Me]GlcAGal_2_.

**Supplemental Figure 5. Measurement of etiolated hypocotyl length.**

Wildtype and mutant seedlings were grown in the dark under etiolation conditions for nine days. Mutants do not show a hypocotyl growth phenotype compared to Wildtype plants. Bars represent SD of 3 biological replicates. No significant differences were identified using two-tailed T-test analysis comparing mutant genotypes to wild type, p<0.05.

**Supplemental Table 1. Percentage of protein sequence identity and similarity among DUF579 family members.**

BLAST-based percentage of identical or similar amino acids among the Arabidopsis DUF579 family members.

**Supplemental Literature Cited**

**Ciucanu I, Kerek F** (1984) A simple and rapid method for the permethylation of carbohydrates. Carbohydrate Research **131:** 209-217

**Gibeaut DM, Hulett J, Cramer GR, Seemann JR** (1997) Maximal Biomass of Arabidopsis thaliana Using a Simple, Low-Maintenance Hydroponic Method and Favorable Environmental Conditions. Plant Physiology **115:** 317-319

**Haghighat M, Teng Q, Zhong R, Ye ZH** (2016) Evolutionary Conservation of Xylan Biosynthetic Genes in Selaginella moellendorffii and Physcomitrella patens. Plant Cell Physiol **57:** 1707-1719

**Li X, Jackson P, Rubtsov D, Faria-Blanc N, Mortimer J, Turner S, Krogh K, Johansen K, Dupree P** (2013) Development and application of a high throughput carbohydrate profiling technique for analyzing plant cell wall polysaccharides and carbohydrate active enzymes. Biotechnology for Biofuels **6:** 94

**Proost S, Van Bel M, Vaneechoutte D, Van de Peer Y, Inze D, Mueller-Roeber B, Vandepoele K** (2015) PLAZA 3.0: an access point for plant comparative genomics. Nucleic Acids Res **43:** D974-981

**Schmid M, Davison TS, Henz SR, Pape UJ, Demar M, Vingron M, Scholkopf B, Weigel D, Lohmann JU** (2005) A gene expression map of Arabidopsis thaliana development. Nat Genet **37:** 501-506

**Schneider CA, Rasband WS, Eliceiri KW** (2012) NIH Image to ImageJ: 25 years of image analysis. Nat Methods **9:** 671-675

**Sparkes IA, Runions J, Kearns A, Hawes C** (2006) Rapid, transient expression of fluorescent fusion proteins in tobacco plants and generation of stably transformed plants. Nat Protoc **1:** 2019-2025

**Tryfona T, Liang H-C, Kotake T, Kaneko S, Marsh J, Ichinose H, Lovegrove A, Tsumuraya Y, Shewry PR, Stephens E, Dupree P** (2010) Carbohydrate structural analysis of wheat flour arabinogalactan protein. Carbohydrate Research **345:** 2648-2656

**Tryfona T, Liang H-C, Kotake T, Tsumuraya Y, Stephens E, Dupree P** (2012) Structural Characterization of Arabidopsis Leaf Arabinogalactan Polysaccharides. Plant Physiology **160:** 653-666

**Tryfona T, Stephens E** (2010) Analysis of carbohydrates on proteins by offline Normal-Phase Liquid Chromatography MALDI-ToF/ToF-MS/MS. Methods in Molecular Biology **658:** 137-151

**Wee EG, Sherrier DJ, Prime TA, Dupree P** (1998) Targeting of active sialyltransferase to the plant Golgi apparatus. Plant Cell **10:** 1759-1768
