# Supplementary material for: Two members of the DUF579 family are responsible for arabinogalactan methylation in Arabidopsis
Source: Plant Direct. 2019 Feb 12;3(2):e00117. doi: 10.1002/pld3.117 (PMC6508755; doi:10.1002/pld3.117)
Supplement: Supplementary file 2 [file PLD3-3-e00117-s002.pdf]

Editorial Comments on first submission

> Thank you for submitting to Plant Direct. The editorial board has
> evaluated your manuscript and concluded that further revisions are
> needed before the paper can be accepted for publication in Plant
> Direct. Specifically, you are being provided with two options. One
> option is to provide biochemical evidence on the methyltransferase
> activity of two AGMs. The other option is to provide more complete
> analysis of mutant lines (i.e. agm2) and tone town your claims on the
> in planta methyltransferase activity of these two enzymes. Without
> direct biochemical evidence, it would be inappropriate to claim that
> methyltransferase activity for these proteins have been confirmed. So
> statements along those lines should be modified.

Response:

We have opted for the second option because our attempts at in vitro reactions were unsuccessful for technical reasons.

We have toned down the claims for the methyltransferase activity. Specifically, the title, abstract and text have been altered to indicate the AGMs are necessary for methylation. We suggest this is strong evidence for their activity, but we do not state we have shown the activity.

Second, we have grown and analysed the mutants again including the *agm2* single mutant. The data show that there is little change in methylation in the agm2 single mutant, but the agm1 agm2 double mutant has lost the methylation of the AGP oligosaccharides tested.
